# Problems and needs for improving primary care of osteoarthritis patients: the views of patients, general practitioners and practice nurses

**DOI:** 10.1186/1471-2474-7-48

**Published:** 2006-06-02

**Authors:** Thomas Rosemann, Michel Wensing, Katharina Joest, Matthias Backenstrass, Cornelia Mahler, Joachim Szecsenyi

**Affiliations:** 1 Department of General Practice and Health Services Research, University of Heidelberg, Voβstr. 2, 69115 Heidelberg, Germany; 2 Center for Quality of Care Research (WOK), Radboud University Nijmegen Medical Centre, P.O. Box 9101, 6500 HB Nijmegen, The Netherlands; 3 Center of Psychosocial Medicine, Clinic of Psychiatry, University of Heidelberg, Voβstr. 4, 69115 Heidelberg, Germany

## Abstract

**Background:**

Osteoarthritis (OA) is highly prevalent and has substantial impact on quality of life as well as on healthcare costs. The general practitioner (GP) often is the first care provider for patients with this chronic disease. The aim of this study was to identify health care needs of patients with OA and to reveal possible obstacles for improvements in primary care management of OA patients.

**Methods:**

We performed semi-structured interviews with a stratified sample of 20 patients, 20 GPs and 20 practice nurses.

**Results:**

Diagnosing OA posed no major problem, but during the course of OA, GPs found it difficult to distinguish between complaints resulting from the affection of the joints and complaints related to a concomitant depression. Patients felt to be well informed about the degenerative nature of the disease and possible side effects of medications, but they lacked information on individual consequences of the disease. Therefore, the most important concerns of many patients were pain and fear of disability which they felt to be addressed by GPs only marginally. Regarding pain treatment, physicians and patients had an ambivalent attitude towards NSAIDs and opiates. Therefore, pain treatment was not performed according to prevailing guidelines. GPs felt frustrated about the impact of counselling regarding life style changes but on the other hand admitted to have no systematic approach to it. Patients stated to be aware of the impact of life style on OA but lacked detailed information e.g. on how to exercise. Several suggestions were made concerning improvement.

**Conclusion:**

GPs should focus more on disability and pain and on giving information about treatment since these topics are inadequately addressed. Advanced approaches are needed to increase GPs impact on patients' life style. Being aware of the problem of labelling patients as chronically ill, a more proactive, patient-centred care is needed.

## Background

About 10% of men and 18% of women aged 60 years and over suffer from symptomatic osteoarthritis (OA) [1]. Due to increasing life-expectancy as well as constant increase in the average Body-Mass-Index, which constitutes a principal risk factor for OA [2], the incidence of OA is expected to rise in years to come [3]. OA causes high direct costs (0.7 % to 1.2 % of the gross national product), and also high indirect costs as a consequence of morbidity and disability and represents one of the diseases with the highest economic impact [4-7].

The main care provider for many patients with OA is the GP [8,9]. To improve quality of care for osteoarthritis patients, evidence based guidelines and a quality indicator set exist in most countries, this, however, is not the case in Germany [10,11].

It has been suggested that conservative management of OA is difficult for physicians and patients, but little is known about the actual management of OA in primary care in Germany. Moreover, there is only limited information about the perspectives of different groups involved in the treatment of OA [12]. For instance, practice nurses play an increasing role in providing care in many health care systems, but their perspective is often ignored [13,14]. Insight into patients', physicians' and practice nurses' views on management of OA is needed to identify problems concerning quality of care and possibilities for improvement. The aim of our study was to reveal patients' needs, assess their wish for improvement and to identify obstacles that handicap improvements. In order to implement such improvements, it is important to not only assess the views of patients, but also those of doctors and practice nurses. The interview guideline was created according to our hypothesis that patients lack information on the disease, medication and possible approaches and wish for more consultation time. The results of this study should help create interventions for the primary care setting, evaluated in a subsequent intervention study.

## Methods

A qualitative interview study with general practitioners (GPs), practice nurses and patients with OA was performed according to the guidance for qualitative research [15]. A heterogeneous sample of 20 GPs, 20 practice nurses and 20 patients was stratified by gender and urbanisation level [16]. The GPs were to have a minimum of 5 years experience; the assistants were required to have a minimum of 10 years professional experience. The patients were selected at random from the GPs'computer files by searching for patients with the ICD -code M16.0–16.9 (coxarthrosis) and M17.0–17.5 (gonarthrosis). During their practice visit they were asked by the GP if they wanted to participate in an interview. All patients but one agreed to participate. The study protocol (named as "Praxarth-barriers-study") has been approved by the Ethical committee of the University of Heidelberg; approval number: 019/2004.

### Interviews

After a detailed study of the literature on evidence-based, non-surgical treatment options for OA and regarding patient perspectives in chronic diseases, we compiled a semi-structured interview guide with open-ended questions. All interview guidelines were as similar as possible to allow comparisons across groups and followed the normal course of a consultation: diagnostic routines, information giving, prescribing, advices for a lifestyle change and referral. Due to the small number of non-surgical evidence based treatment options we were especially interested what importance evidence based treatments have. In addition, we focused on the attitudes of patients, doctors and assistants towards a larger involvement of the practice nurse in the care of patients suffering from OA.

### Procedures

The interviews were conducted during 2004. The GPs and practice nurses were interviewed in their respective practices; the patients were interviewed at home by trained interviewers. During the interview, the interviewer ensured that every aspect was explained sufficiently and in detail, so that there would be no questions or misunderstandings later on.

### Analysis

The conversations were recorded digitally, transcribed literally and analysed by four different researchers with ATLAS.ti-Software [17]. An initial categorising system was established based on the interview guidelines. In order to achieve maximum objectivity, all interviews were read by four researchers and categorised independently. The categorising system was consequently modified; subcategories were added after agreement had been reached among all four researchers. Numerous free categories were developed from the text, discussed and adjusted in an iterative process so that they were as similar as possible in all three interviewed groups, as the objective was to emphasise the different perspectives of the groups regarding individual subject complexes. The codes were clearly defined and linked with representative examples from the original text.

## Results

The mean age of our patients was 56, with a range from 40 to 78 years as can be seen in table 1. The educational level was relatively high. Working experience ranged from 8–19 years with a mean of 11.3 years among GPs and from 13–35 years (mean: 21.7) among practice nurses. Some items yielded very limited responses among practice nurses, therefore their statements were only mentioned if they provided an important contribution to a specific aspect. Tables 2, 3, 4 display the categorical system with subcategories. The numbers in brackets display how many participants responded to the respective category.

### Diagnotic aspects – proceedings

The interviewed GPs stated that in most cases diagnosing OA poses no major problem to them. The diagnosis is frequently based on an extensive anamnesis and an accurate examination. The interviewed GPs stated that if they are unsure whether the pain is caused by the joint or periarticular structures, an x-ray is performed to confirm OA. During the course of OA, the situation is more difficult: it sometimes represents a challenge for GPs to distinguish between complaints resulting from the joint affection and complaints which are mainly related to depressed mood. Satisfaction among patients regarding the diagnostic procedure was high: most patients in our study sample stated that the GP took enough time in diagnosing and that the examination was extensive and accurate.

When asked about how and to what extent GPs inform patients about the disease, some GPs stated that they try to assess the patients' need for information and their capability to understand, but also what they assume the patient can handle. Overall, patients were considered to be well informed due to their utilisation of countless other sources of information such as print media and TV.

This assumption was confirmed by many patient statements. Regarding the cause and the pathomorphology, patients felt well informed. Most of them were aware of the degenerative nature of the disease. There was no apparent lack or request for more information on this topic. But in terms of the prognosis, patients were very insecure. Pain and becoming disabled were the main fears of patients and most of them stated that they were insecure to what extent pain could increase and if they would be able to walk at some point in time. Many patients argued that physicians were mainly focused on explaining the pathology of the disease and the treatment options such as new surgical methods, but less focused on their main fears. Especially older patients seemed to have problems mentioning these concerns. In conclusion, there was no quantitative lack of information, but a qualitative one, as the following two statements reflect:

*"The majority of patients nowadays open the envelope (of the specialist) themselves. They know exactly what is written down there. There is communication on the same level between doctor and patient. " *(GP 10, male, 37 years)

*"I know that OA is a one-way-street. That's not the problem. Life is a one-way-street too. But in OA I don't really know what's at the end." *(Patient 17, male, 71 years)

### Diagnotic aspects – problems

Asked about problems in the diagnostic process, most of the interviewed GPs were aware that like many other diseases of the musculoskeletal apparatus, OA only shows little correlation between what is pathomorphologically visible – e.g. on a radiographic image – and subjective complaints. Therefore, many GPs stated that they found it difficult to assess to what extent complaints originate from arthritis and what part of the complaints are due to concomitant depressive symptoms. This was particularly the case when there was unsufficient radiographic evidence and the physical examination gave no sign for an acute inflammation of the joint. Depression was also recognized as an important barrier to motivate patients to physical exercise. Concrete instruments, such as well known questionnaires for instance the HAMDS [18] or the PHQ-9 [19] etc. were not used to reveal depression. One GP stated:

*"And there is always a depressive component. The relation between depression and arthritis pain and physical sensation is an important one. And exactly those people with depression cannot change anything about it, because they really suffer from depression and are not capable of changing their lives or doing something about their lives; they fall deeper and deeper into this vicious circle of disease and pain, and nobody can help them." *(GP 5, male, 47 years)

According to GPs' statements, and confirmed by most patients of our sample, nearly every patient was sooner or later referred to an orthopaedic surgeon in order to confirm the diagnosis by taking an x-ray.

*"He was more interested in taking pictures of my knee than in examining it"*. (Patient 19, female, 68 years about an orthopaedic specialist)

Patients regarded specialists as an additional source of information, but most of them mentioned that the GP took definitely more time for the anamnesis and was often more accurate than the specialist. Many patients stated orthopaedics would be mainly interested in the use of machines then in talking to them. GPs also had an ambivalent attitude towards these referrals. On the one hand they did not recognize superiority in knowledge and treatment options of a conservative treatment by an orthopaedic surgeon. On the other hand they used the orthopaedic surgeon from time to time to escape from the psychological burden induced by the patient and the absence of treatment options. In addition they felt a lot of pressure by patients to refer them to the specialist, especially in case of younger and well educated patients. But some stated that they sometimes felt abused by patients as well as by specialists, because the patients pushed them to be referred and the specialist did not take the time to explain what they had examined or the x-rays he had taken. Therefore GPs often ignored the patients' repeated requests for referrals to an orthopaedic specialist.

Interestingly, lack of time could not be revealed: most GPs stated to take as much time as possible and patients did not regard time limitation as a main problem or at least showed understanding for the limitation in the face of overcrowded waiting rooms.

### Treatment aspects – pharmacological treatment

Regarding treatment aspects, pharmacological treatment was the topic on which the most statements were recognized, indicating the importance of this topic for all groups. Facing decreasing financial resources and increasing restrictions by most health insurances, many GPs stated that treatments like massages, physiotherapy and manual therapy were prescribed less frequently. Some GPs complained that in consequence, OA treatment has mainly been reduced to prescribing pain medication.

Asked about adherence to guidelines, which recommend Paracetamol as first choice of pharmacological treatment [20-22], GPs stated that Paracetamol was not accepted as a real pain reliever because it is known to most patients as medication for "headache" and available without prescription. GPs also argued that most patients have already taken this drug on their own by the time they visit their physician.

Consequently, Paracetamol was prescribed less by the interviewed GPs and for all of them NSAIDs represented the main pillar in their pharmacological therapy of OA. But after the withdrawal of most COX-2-inhibitors, patients as well as doctors felt very uncertain what to consider as an appropriate pharmacological treatment.

Interestingly enough, patients and GPs have a similar ambivalent attitude towards analgesics. Patients appreciated the alleviation of pain, but at the same time they instinctively rejected these drugs without an apparent rational explanation. For instance, Diclofenac's stomach-irritating potential is such a well known fact that positive aspects of the drug are being ignored. No patient stated that he would take pain reliever in advance; they normally wait until they can not take the pain any longer. GPs felt that due to the package inserts patients mainly focused on side effects and therefore these leaflets were regarded as a barrier for optimal treatment compliance. GPs' main aim was to ensure that the patient actually took the prescribed drugs. Therefore they had mostly developed individual strategies that consisted of a balancing act of explanations for anticipated objections regarding treatment, legal requirements and belittlement.

Asked about the meaning of package inserts, most of the patients stated that information on side effects was not that important to them, because they were aware that many of the side effects mentioned on the package insert never occurred. On the other hand they generally read the package inserts. Most patients of our sample stated that they mostly trusted the information given by their GP. But it seemed that the package inserts alerted them for possible side effects. In conclusion patients as well as doctors are more focussed on side effects then on positive effects such as the anti-inflammatory potential of NSAIDs.

The following statement displays the strategy of one GP in dealing with side effects:

*"Well, my personal opinion is, if you give the patients two or three side effects, they are happy, otherwise they have all of them since they are printed on the package insert. For that reason, I limit myself to two or three that I mention. Sometimes, when you mention it and say,"oh you could get that, but not really, only a few get that", I always attach a negative example, "but I think you are quite fit and healthy, so that you will not get it", then they don't get it*. (GP3, male, 51 years)

Regarding opiates, similar barriers could be revealed from the doctors' as well as from the patients' perspectives: GPs stated that many patients would reject these "heavy drugs" (GP 6) and it seemed that even GPs regarded use of these drugs as overtreatment in OA. Furthermore, most physicians stated not to prescribe them as they are poorly tolerated and cause nausea. Opiates were often recognized by patients as medication for people in very poor condition as e.g. cancer patients and therefore rejected. None of the patients received a structured pain treatment plan or systematic advice to cope with pain. The following statement of a patient (female, 76) reflects a quite typical statement:

*"But I am careful; if I can take the pain then I won't take a pill because they are not really good for you. Only if there is no other way, then I will take one and that has to be enough....I really only take a pill when I am in terrible pain, otherwise I am against drugs." *(Patient 4, female, 71 years),

### Treament aspects – advice giving and counselling

This topic received the second most statements from GPs and patients. Nearly all interviewed GPs emphasised that they repeatedly addressed behaviour interventions that can slow down the progress of OA, including weight loss and the strengthening of musculature. However, most of them admitted that they did not focus on increasing patients' motivation for behavioural change, but just gave general recommendations. The success rate in motivating patients was considered too low by the GPs, and the majority appeared distinctly resignated regarding their impact on patients' life style. Many GPs also mentioned that there was a vicious circle: due to pain when exercising, people move less and eat more due to accompanying frustration and sometimes depression. Being asked about the reasons why it is so hard to communicate these secondary preventive measures to patients, most GPs answered as GP 17 (male, 54 years):

"Osteoarthritis is ultimately only a symptom of a huge lifestyle problem a complete change in lifestyle is required.... and this is impossible for osteoarthritis patients who are mostly elderly people...nobody is willing to change his/her lifestyle due to osteoarthritis, the disease has to be a lot worse than this. People have basically learned to live with it. "

The patients in our sample confirmed GPs' statements regarding life style interventions. The majority indicated that their GP had tried to motivate them repeatedly and had explained the general effects of lack of exercise and overweight. The following statement displays this quite impressively:

*"He really talked to me again and again, once he even asked if I wanted to eat myself into a wheelchair. And if I don't do it then it is my fault. The spirit is willing, but the flesh is weak. " *(Patient 9, female, 68 years)

According to patients' statements, concrete types of exercise or other possibilities were not mentioned, directions were mostly quite vague. Asked about reasons for failure regarding their own physical activity, the patients mentioned pain, lack of knowledge regarding respective offers, lack of mobility and a lack of motivation. Indeed, most of the interviewed GPs stated that they did not inform patients about self-help groups or about offers on community level for instance. Reasons for this were a lack of information and frustration about the impact of this information: The GPs who had experience in giving this information complained that a lot of patients always find excuses not to participate in these services, such as the distance from their homes to the location etc. Contrary to these statements, patients welcomed basic information on self-help groups, but they were often unsure about possible benefits and also expressed their reservations, in particular regarding availability or location in the rural environment. Receiving just a short, vague hint without a clear advice or motivation was regarded as insufficient.

### Suggestions concerning improvement of care

The interviewed GPs were convinced that a gate keeper role for GPs as in many other health care systems could reduce patients' pressure to refer to orthopaedics and decrease performed x-rays. Some GPs mentioned that better communication with specialists could increase efficacy of treatment, but no specific suggestions how to achieve this were made. Many GPs stated that the payment system has to be changed in order to upgrade conservative treatments and conversation with the patient. Due to the insecurity regarding NSAIDS, some GPs also desired evidence based pharmacological recommendations. Interestingly, patients could define their needs of care but ideas for improvement were quite vague such as better communication etc.

For most GPs an involvement of practice nurses -which currently is only marginally the case in Germany- is imaginable in the area of life style counselling and advice giving. Involvement in the diagnostic process was refused. Main barriers mentioned were lack of professional knowledge and lack of time due to administrative overload. Moreover, all GPs stated that interventions performed by practice nurses have to be reinsured sufficiently. Interestingly, practice nurses' opinions were quite similar to GPs' statements: They mentioned lack of knowledge due to professional education which is mainly focused on administrative issues. Especially younger practice nurses desired more involvement. They regarded this as an upgrade of their profession. Some of the nurses declared that they would like to offer links to self-help groups or sport groups if this information would be available in the practice.

To receive information and advices from practices nurses – by printed information or lectures – was acceptable for most patients. But some of them indicated – as some GPs did – that they fear a worsening of the trustful doctor-patient-relationship if the nurse is involved in too many proceedings. However, missing information about offers e.g. in the community, caused statements as the following one:

*"There is a "Nordic walking group" in town...I know that some of our patients participate, but I really don't know to whom I should send the patients to." *(Practice nurse 7, 29 years)

## Discussion

In addressing different areas of OA treatment, our study provides several important findings: the main finding is that although patients with osteoarthritis report on pain and disability as a primary concern, they do not feel that these topics are adequately addressed by their GP or specialty physician. Former studies also revealed a strong desire of OA patients for more information, but it remained unclear what kind of information was mainly required [23]. The qualitative approach of this study helped to specify the patient needs, which are clearly focused on the individual perspectives regarding pain and mobility rather than on information about the pathology of the disease.

Regarding diagnosis and handling of OA, statements of GPs are concordant with previous studies showing that GPs have developed individual approaches to the management of OA. They perceived no major problems in diagnosing OA but had [24,25] difficulties in assessing concomitant depression. Possible implications for practice could be to provide easy-to-use and less time consuming screening tools for depression as e.g. the PHQ-9. Moreover, most GPs seem to be aware that OA in primary care is mainly a syndrome and that x-rays contribute less to the management but may label the patient as chronically ill. As Bedson et al. discussed, this approach may be inadequate in primary care [26]. But many GPs felt urged by patients to perform referral and, consequently the wish for a gate keeper role – which was assumed to reduce this pressure – was frequently mentioned.

In accordance with former research, NSAIDs represented the most important treatment for the interviewed GPs, but also an important source of uncertainty on both sides. Furthermore, ineffective pain treatment is still an important problem on both sides [27-29].

Since Paracetamol is known to be as effective as NSAIDs for mild and moderate OA but associated with fewer side effects than NSAIDs, our findings suggest that GPs' awareness about this fact needs to be increased. They should also communicate this to their patients. But also if NSAIDs or opiates are required, positive effects of NSAIDs and opiates and importance of pain control for physical ability instead of arguing about side effects could lead to a more appropriate pain treatment.

GPs considered their impact on life style of patients as low and were quite frustrated about behavioural interventions. Appropriate motivation strategies and lectures on adequate sport for patients could be possible interventions. Ideally, these educational activities are connected with a linkage to local patient groups and community offers. The practice nurse could provide advice to individual patients or groups of patients (similar to the already existing sessions with diabetes patients), provide follow-up by telephone to support behaviour change in patients, and provide information on community support. All these options imply new roles of the practice nurse in Germany, so evaluations to test the feasibility and effectiveness of these roles are recommended.

GPs desired a gate keeper role to decrease patients' pressure for extensive diagnostic procedures and referrals. Involvement of practice nurses were considered reasonable in advice giving and life style counselling.

Our study was probably the first to simultaneously examine the perspectives of primary care physicians, patients and practice nurses on the management of osteoarthritis simultaneously. We noticed that patients in our study were relatively old. Older people tend to be happier with the health care they receive [30,31]. On the other hand, our study sample was consistent with the real patient population suffering from osteoarthritis in primary care. Nevertheless some limitations have to be considered. The aim of qualitative research is to generate ideas and hypotheses. Due to the methodological approach and the sample size, quantitative conclusions can not be drawn. It is also important to recognize that the statements reflect individual opinions, and that e.g. self reported behaviour must not correctly reflect the real behaviour or does not reflect reality. For instance, if GPs report they have no problem in distinguishing articular form periarticular pain this does not mean that they are correct in doing so. Additionally, the German system of care of people suffering from musculoskeletal disorders may be unique in the world due to the high amount of non-surgical orthopaedic physicians working in practices and representing some kind of midlevel structure between primary care and the orthopaedic surgeon located at hospitals. Problems arising from this situation, as for instance the high frequency of performed referrals and x-rays can not easily be transferred to different health care systems.

## Conclusion

Osteoarthritis is a disease which will become increasingly visible in years to come. In search of practical and simple interventions on a primary care level, this study resulted in a series of valuable suggestions about what patients require and how a practice team can respond: GPs should focus more on disability and pain and on giving information about treatment since these topics are often inadequately addressed. Advanced approaches are needed to increase GPs' impact on patients' life style. Being aware of the problem of labelling patients as chronically ill, a more proactive, patient-centred care is needed.

## Competing interests

The author(s) declare that they have no competing interests.

## Authors' contributions

TR and MW conceived and performed the study and draft the manuscript. CM draft the manuscript, KJ and MB were involved in the development of the questionnaire and the data analysis with ATLAS.ti. All authors read and approved the final manuscript.

## Funding

This study was part of the PRAXARTH project that aims to improve the quality of life of patients suffering from OA. The project is financed by the German Ministry of Education and Research (BMBF), grant-number 01GK0301.

## Pre-publication history

The pre-publication history for this paper can be accessed here:



## Figures and Tables

**Table 1:** Baseline characteristics of the study sample

	N (female)	Age (mean)	Years of working experience (mean)	Education level * (mean)	Interview time (minutes)
practice nurses	20 (20)	29–56 (41.3)	13–35 (21.7)		25 (21–34)
GPs	20 (4)	33–57 (43.5)	8–19 (11.3)		45 (41–60)
patients	20 (12)	40–78 (56.2)		3.3	43 (41–55)

* (1 = no school; 5 = university degree)

**Table 2:** Main categories regarding diagnostic aspects

**General Practitioners**	**Patients**	**Practice Nurses**
***Proceedings*^a^*(19)*^b^:** Making an extensive anamnesis^c ^(19) Making an extensive examination (18) Informing patients about cause and course of disease (17) Referring patients to orthopeadic (8) Referring to radiologist (6) ***Problems*** -*diagnosing OA (12):* Poor correlation between x-rays and complaints (8) Concomitant depression (5) Others (3) -*regarding specialist (6)* Focusing on performing x-rays (5) Missing information about performed examinations/recommendations (5) Felt pressure to refer to specialist (3)	***Satisfaction (20):*** Satisfied with diagnostic proceedings (15) ***Needs/problems regarding GPs (13****)* Missing information about diagnosis and its course/impact on individuals life (12) Lacking time (2) ***Needs/problems regarding specialists (15)*** Lacking time (14) Missing information about diagnosis and its course/impact on individuals life (8) Focussing on performing examinations (5) Recommendation of expensive treatments (4)	***Involvement (20):*** Current involvement in - diagnostic proceedings (2) - treatment (1) No involvement in diagnostic proceedings (17) Wish of being more involved (11) No wish of being more involved (9) ***Barriers against involvement (20)*** Lacking knowledge about disease (13)/treatment (17) Lacking time due to - administrative overload (11) - other reasons (3)

^a ^Words in italic are main categories; ^b ^number of participants referring to the respective category (multiple mentions possible)

^c ^subcategories (not all subcategories are displayed).

**Table 3:** Main categories regarding treatment aspects

**General Practitioners**	**Patients**	**Practice Nurses**
***Applied (non-surgical) treatments (20):*** Prescribing Pain relievers (19) Prescribing physiotherapy/massages (3) Counselling (19) - to reduce weight (19) - to exercise (15) - others (2) ***Problems (20):*** *General (14):* - Increasing restrictions regarding prescription of physiotherapy, etc. (14) - Lack of time (3) *Regarding pharmacological treatment (20)*: - Package leaflets decrease adherence (14) - Insecurity with pharmaco-logical treatment/guidelines (12) - Little acceptance of opiats (6) - Interaction with specialist (5) *Regarding life-style:* - Motivating the patient to exercise or reduce weight (17) - Concomittant depression (6)	***Needs/Expectations (20):*** Improvement of symptoms - pain (14) - physical activity (8) Pointing out possible treatments (5) Conversation about the problems (2) Referral (2) ***Problems (20):*** *Regarding medication (20):* - (Fear of) side effects (16) - Lacking pain relief (12) *Regarding counselling (15):* - reasons laying in individual behaviour (14) - no precise advices to reduce weight/exercise (8) - lacking information about supportive offers (6)	***Knowledge (20):*** Knowledge about treatment (18) No Knowledge about treatment (2) ***Involvement (20):*** Involvement in treatment (2) No Involvement in treatment (18) Wish of being more involved (14) No wish of being more involved (5)

**Table 4:** Suggestions concerning improvement of care

**General Practitioners**	**Patients**	**Practice Nurses**
***Ideas how to improve treatment (19):*** Gate keeper role for GP (14) Financial reward for communication/more time for conversation (11) Evidence based pharmacological recommendations (5) Improved cooperation with orthopaedics (5) Integrating the patients' social system (e.g. family) into treatment (2) More openly address psychological complaints of the patients (1) ***Team approach (20):*** Imaginable (18) Not imaginable (2) ***Possible tasks for practice nurses (20):*** Information about offers on the community level/self help groups (11) Providing additional written information (8) Relaxation techniques (2)	***Whishes concerning GP (16):*** Transparency concerning normal course of disease (11) Using less medical terms (3) GP should more openly address psychological complaints, direct questions about mood (3) More time (1) ***Team approach (20):*** Imaginable (13) Not imaginable (7) ***Possible tasks for practice nurses in the context of a team approach (10):*** Informing the patient about additional (non-pharmacological) approaches (9) Informing about offers on the community level, sport groups, self help groups (7) Asking for side effects (5) Asking patient about his mood (4) Talking to the patient sympathetically (3)	***Team approach (20):*** Imaginable (15) Not imaginable (5) ***Possible tasks for practice nurses in the context of a team approach (15):*** Talking to the patient (9) Counselling in groups (2) Asking patient about reason for consultation (2) Calling the patient in regular intervals and ask about pain, side effect of medications (2) Motivating the patient to use self-help groups and social contacts (2) Organising self-help groups (2) Exchanging information about the patient with the GP (2)
